# Supplementary material for: Explainable machine learning identifies multi-omics signatures of muscle response to spaceflight in mice
Source: NPJ Microgravity. 2023 Dec 13;9:90. doi: 10.1038/s41526-023-00337-5 (PMC10719374; doi:10.1038/s41526-023-00337-5)
Supplement: Supplementary file 1 — Supplementary Information [file 41526_2023_337_MOESM1_ESM.pdf]

Explainable machine learning identifies multi-omics signatures of muscle response to spaceflight in mice

Authors

Kevin Li[1,2*], Riya Desai[3*], Ryan T. Scott[1,9], Joel Ricky Steele[4,8,9], Meera Machado[5], Samuel Demharter[5], Adrienne Hoarfrost[6], Jessica L. Braun[7], Val A. Fajardo[7], Lauren M. Sanders[8,9], Sylvain V. Costes[9]

1. KBR, Moffett Field, CA, USA
2. NASA Space Life Sciences Training Program, Moffett Field, CA, USA
3. College of Letters and Science, University of California at Davis, Davis, CA, USA
4. Monash Proteomics and Metabolomics Platform, Monash Biomedicine Discovery Institute, Monash University, Clayton, Victoria 3800, Australia
5. Abzu ApS, Denmark
6. Department of Marine Sciences, University of Georgia, Athens, GA, USA
7. Department of Kinesiology, Centre for Bone and Muscle Health, Brock University, Canada
8. Blue Marble Space, Seattle, WA, USA
9. Space Biosciences Division, NASA Ames Research Center, Moffett Field, CA, USA

* Co first author

Supplementary Methods

Dimensionality reduction

Following the preprocessing of the omics datasets, we then applied Multi-Omics Factor Analysis (MOFA) on the combined multi-omic datasets using the MOFA2 v1.4.0 R package[39]. MOFA is an unsupervised dimensionality method that uses Bayesian prior/posterior distribution updating to find a small set of latent factors to capture the variance in the data, which are analogous to principal components in PCA[39]. Unlike PCA, however, MOFA relies on probabilistic principals instead of geometric properties and is able to find its latent factors using information from multiple omics datasets (e.g. a factor is determined based on information provided by the transcriptomic, proteomic, and epigenomic datasets). Additionally, MOFA assumes that the distribution is continuous and roughly Gaussian, which was the case for our normalized and transformed datasets (Supplementary Figure 3, Supplementary Figure 4A,B,E,F).

MOFA Factor 1, which captures most of the variance in the dataset, cleanly separated FLT and GC samples in both datasets (Supplementary Figure 6A,B). We inferred that the features associated with Factor 1 would be most relevant for examining expression differences underlying spaceflight-induced physiological changes; therefore, for each data type, we selected only the top K features associated with MOFA Factor 1 to use for downstream methods.

To derive K, we also performed a Weighted Gene Correlation Network Analysis (WGCNA v1.71)[40] on each data type and extracted features from clusters with significant differences between FLT and GC samples as follows. In WGCNA, all features within each dataset were sorted into clusters based on their respective correlation between FLT and GC groups. The clusters were sorted by correlation, and the features contained in the clusters with the highest correlation (>0.18) were selected. This cutoff was selected so that a sufficient number of features could be selected from each dataset to allow for an ample overlap with MOFA selection. We then chose K to give maximum % overlap between MOFA Factor 1 features and WGCNA features (Supplementary Figure 7), since overlap between important features extracted from two different unsupervised methods increases confidence in the true significance of those features.

After evaluation, we found that the factors found by MOFA were primarily driven by RNA-seq and proteomic features, with very few methylation features (Supplementary Figure 6C,D). Therefore, for methylation feature reduction, we ran MOFA with only the methylation dataset. There were no factors that clearly separated the FLT vs GC, so we selected top methylation features associated with all factors found by MOFA (5 total) whose variance contributions were not driven by outliers (one factor was not included because outlier data points drastically inflated its variance contribution)[7]. WGCNA was finally used to identify methylation features from the top MOFA features that gave highest correlation between GC and FLT.

When feeding features from different omics types into QLattice, all features were automatically set to comparable scales within QLattice using a variation of min-max scaling [7,41].

Cross validation

Due to the small sample size of our dataset, we decided to perform leave-one-out cross validation (LOOCV). During each iteration of LOOCV, the model is trained on the entire dataset except one sample, which is used as a validation sample to test the model's generalizability. This is done for *n* iterations (where *n* is the size of the dataset) so that each sample is used as a validation

sample at some point. Because the training set for each iteration of LOOCV contains only one fewer sample than the full dataset, LOOCV is approximately statistically unbiased[42].

Since QLattice outputs the top 10 model architectures for each run, the 12 iterations of LOOCV generated 120 distinct models, where each was used to predict either the calcium reuptake AUC label (in the regression problem) or the FLT/GC label (in the classification problem) of one validation sample. For the regression problem, we then calculated the multiple R^2 between these 120 validation sample predictions and the corresponding calcium reuptake AUCs; for the classification problem, we calculated the accuracy among these 120 predictions. We call these the T10 validation scores. Additionally, we used the same measurements, but only considering the top model from each iteration instead of the top 10 (12 models total instead of 120); we call these the T1 validation scores. While these validation metrics do not represent the generalizability of a particular model architecture, they do represent the overall ability of QLattice to find generalizable models and thus inform the overall generalizability of biological results derived from all models.

Assignment of Calcium Reuptake Labels

One caveat of our study is that the calcium reuptake measurements were collected from different mice than the omics data. The SOL calcium reuptake measurements were collected from mice from the same RR-1 cohort as the OSD-104 SOL omics data, while the tibialis anterior calcium reuptake measurements were collected from mice flown on the RR-9 mission. We assessed inter- and intra-condition differences in calcium reuptake AUC for both muscle types, and found that the inter-condition differences between FLT and GC mice were much greater than the intra-condition variation, indicating that AUC levels are likely consistent within a cohort of FLT or GC mice. Therefore, for the purposes of relating multi-omics features to overall calcium reuptake levels, we randomly assigned each mouse in OSD-104 and OSD-105 the calcium reuptake AUC of a mouse with the same biological condition and muscle type from OSD-488.

In this process, we found that different random calcium label assignments affected subsequent QLattice validation performance. We generated a distribution of 60 random label assignments and their corresponding QLattice validation multiple R^2 values, and found that one particular labeling assignment for each muscle type resulted in drastically higher QLattice validation performance (Supplementary Figure 8).

Our hypothesis is that each mouse in OSD-104/105 is most physiologically similar to a particular mouse in OSD-488, resulting in an optimal labeling assignment when the calcium reuptake values are assigned to the multi-omics features from the most similar mouse. This hypothesis is supported by the fact that for each muscle type, a single labeling assignment drastically outperformed the others, which suggested that these assignments likely did not outperform just by random chance. Therefore, we chose the optimal labeling assignment for each muscle type for downstream machine learning analyses. The biological relevance of the results (see Results) further increased our confidence that the high validation performance of this labeling assignment was not driven only by spurious correlations.

Various Multi-Omics Combinations

In addition to the QLattice runs mentioned in the paper, we also tried running QLattice on various other omics combinations.

For OSD-104 regression analysis, we tried running QLattice using only the methylation data, since RNA-seq features seemed to dominate the models in the multi-omics runs. These methylation-only models performed very poorly (validation $R^2 < 0.15$), again demonstrating the lack of useful information contained within gene-level methylation data for this specific study.

For OSD-105 regression analysis, we performed additional runs of QLattice using all three omes (RNA-seq + proteomics + methylation) and only RNA-seq + methylation data. The RNA-seq + methylation data performed quite poorly (validation $R^2 < 0.4$), signifying the importance of proteomic features for the TA data. Interestingly, the runs using all three omes, while not performing terribly (T1 and T10 validation R^2 scores of 0.716 and 0.476, respectively), did noticeably worse than the runs using only RNA-seq and proteomics. A potential explanation for this is that the introduction of noisy gene-level methylation features not only doesn't help, but 1) can exacerbate the risk of overfitting and 2) drastically expands the search space for QLattice to explore (this was also suggested when we needed to increase the number of evolutionary epochs QLattice would explore for in order to even get comparable results to the RNA-seq + proteomic only runs). However, despite the lower performance, Acyp1 and Rps7 still dominated in the resulting models when using all three omes.

Supplementary Figures

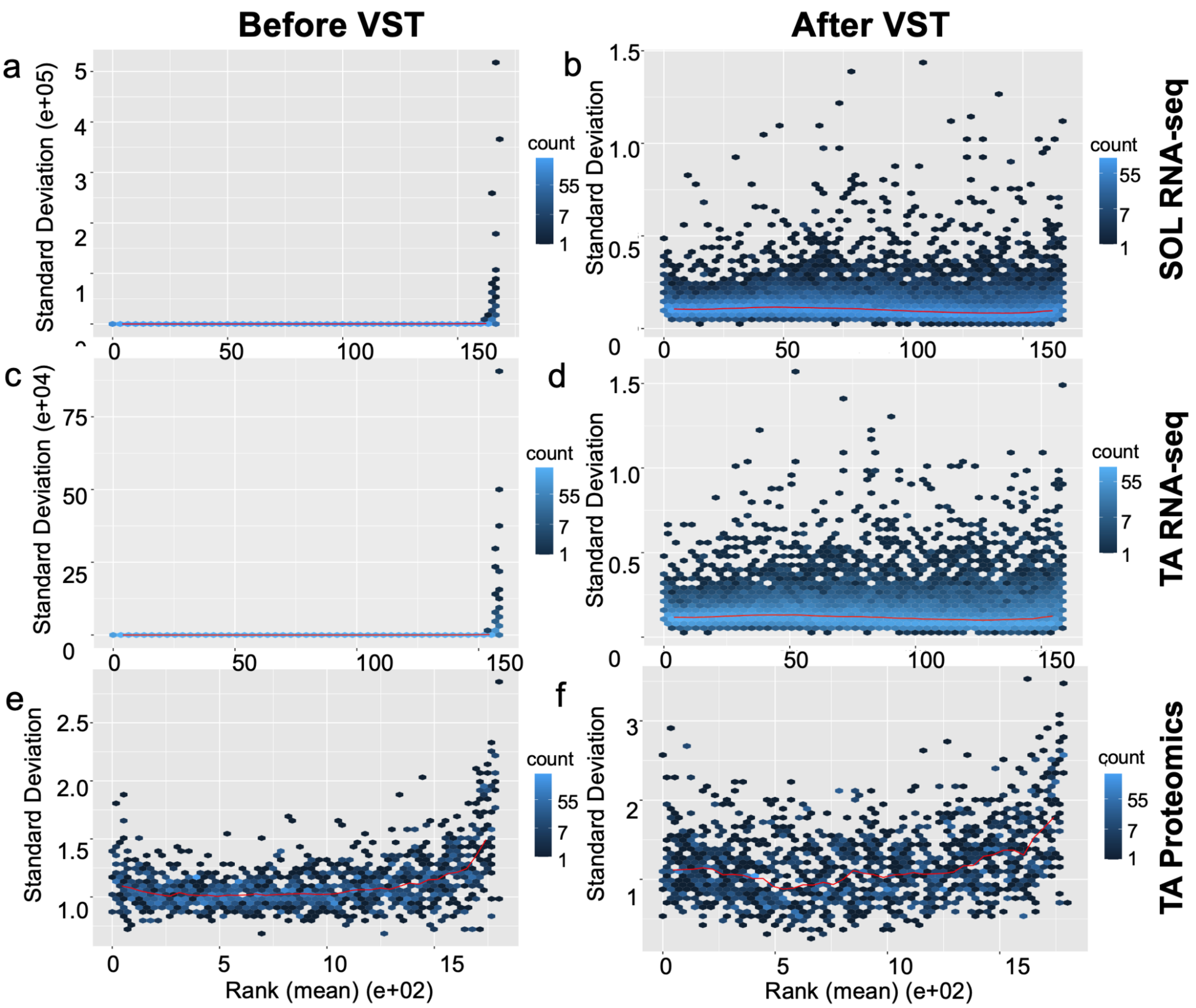

Supplementary Figure 1. Mean-variance relationships for OSD-104 SOL RNA-seq and OSD-105 RNA-seq and proteomics before and after VST. a) Mean-variance relationship before VST for SOL RNA-seq. **b)** Mean-variance relationship after VST for SOL RNA-seq. **c)** Mean-variance relationship before VST for TA RNA-seq. **d)** Mean-variance relationship after VST for TA RNA-seq. **e)** Mean-variance relationship before VST for TA proteomics. **f)** Mean-variance relationship after VST for TA proteomics.

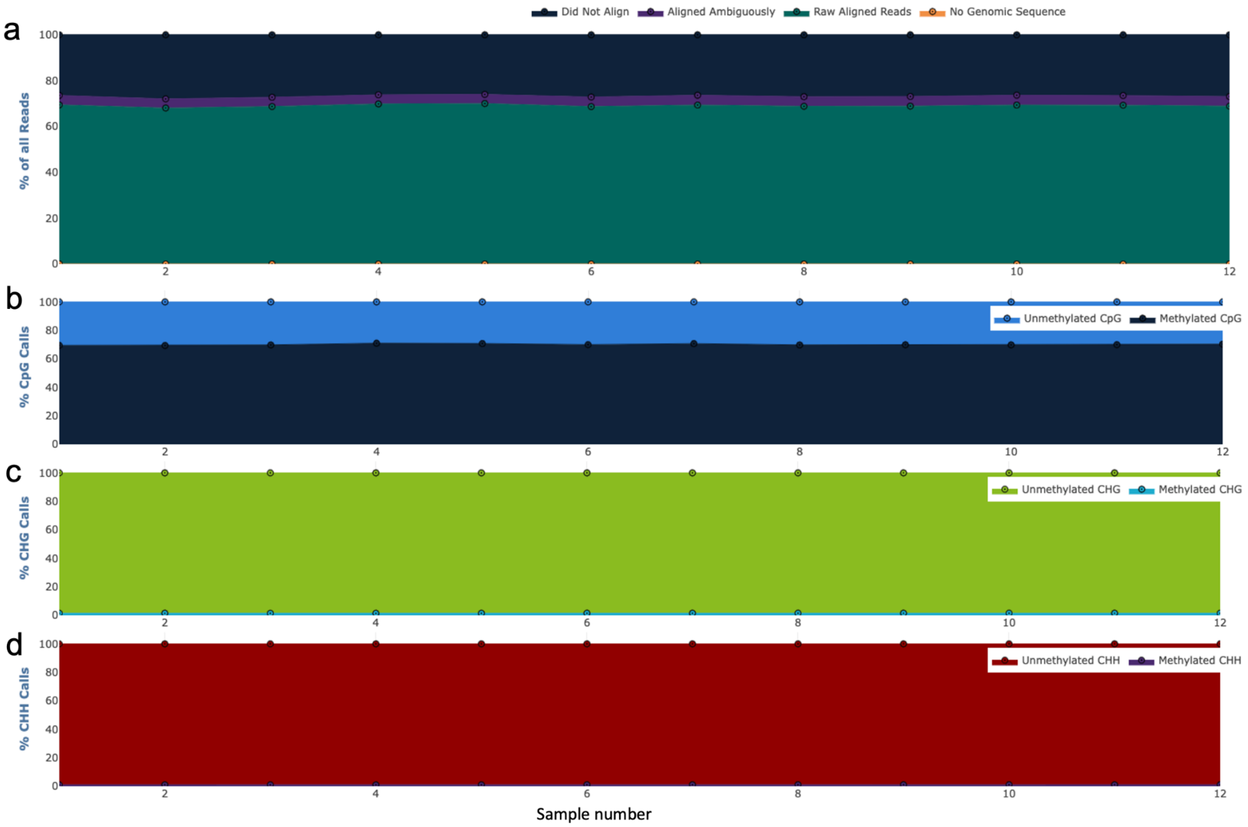

Supplementary Figure 2. Bisulfite sequencing alignment metrics for OSD-105 TA data. a) Percent reads aligned for OSD-105 (TA) raw bisulfite sequencing alignment with Bismark. **b)** Percent CpG calls for OSD-105 (TA) bisulfite sequencing data. **c)** Percent CHG calls for OSD-105 (TA) bisulfite sequencing data. **d)** Percent CHH calls for OSD-105 (TA) bisulfite sequencing data.

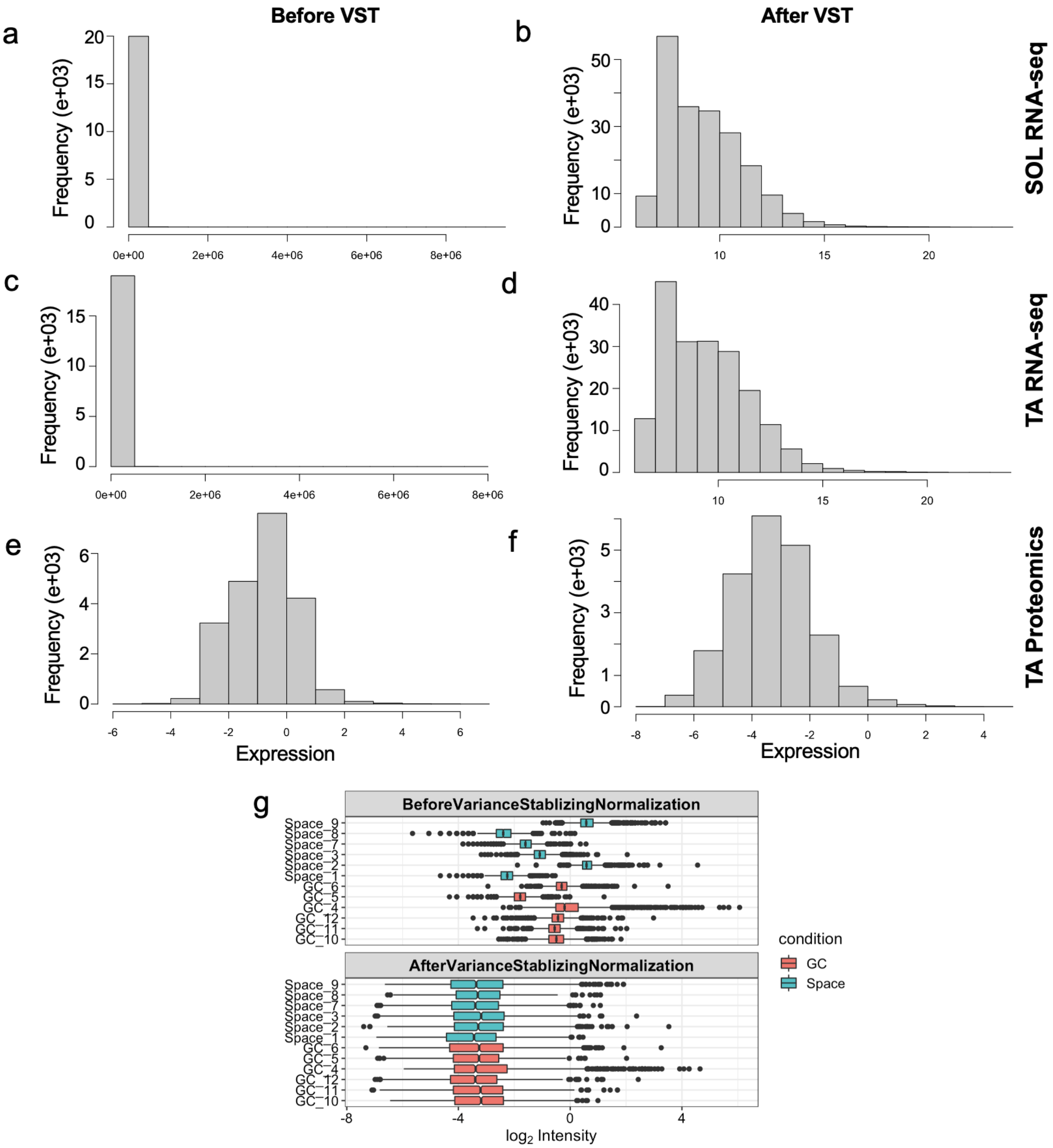

Supplementary Figure 3. Data distributions before and after VST. a) Distribution of normalized expression values before VST for SOL RNA-seq. **b)** Distribution of normalized expression values after VST for SOL RNA-seq. **c)** Distribution of normalized expression values before VST for TA RNA-seq. **d)** Distribution of normalized expression values after VST for TA RNA-seq. **e)** Distribution of normalized expression values before VST for TA proteomics. **f)** Distribution of normalized expression values after VST for TA proteomics. **g)** Distributions of normalized expression values for each sample before and after VST for TA proteomics.

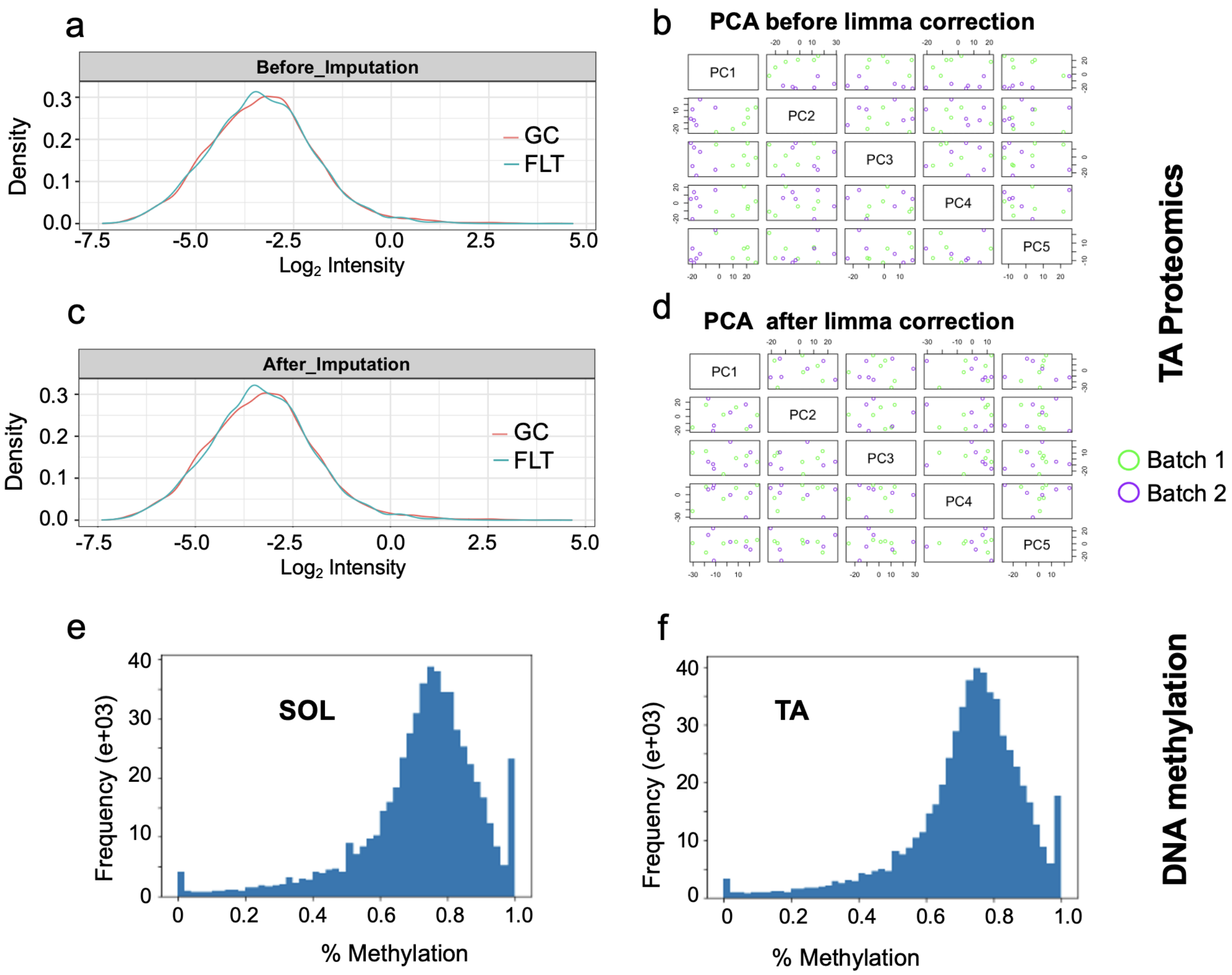

Supplementary Figure 4. Proteomics data distributions before and after imputation and limma and % methylation distributions. a) Distribution of TA proteomics data before imputation. **b)** PCA plots for proteomics data color-coded by TMT run before correction for batch effects. **c)** Distribution of TA proteomics data before imputation. **d)** PCA plots for proteomics data color-coded by TMT run after correction for batch effects. **e)** Distribution of % DNA methylation values for SOL. **f)** Distribution of % DNA methylation values for TA.

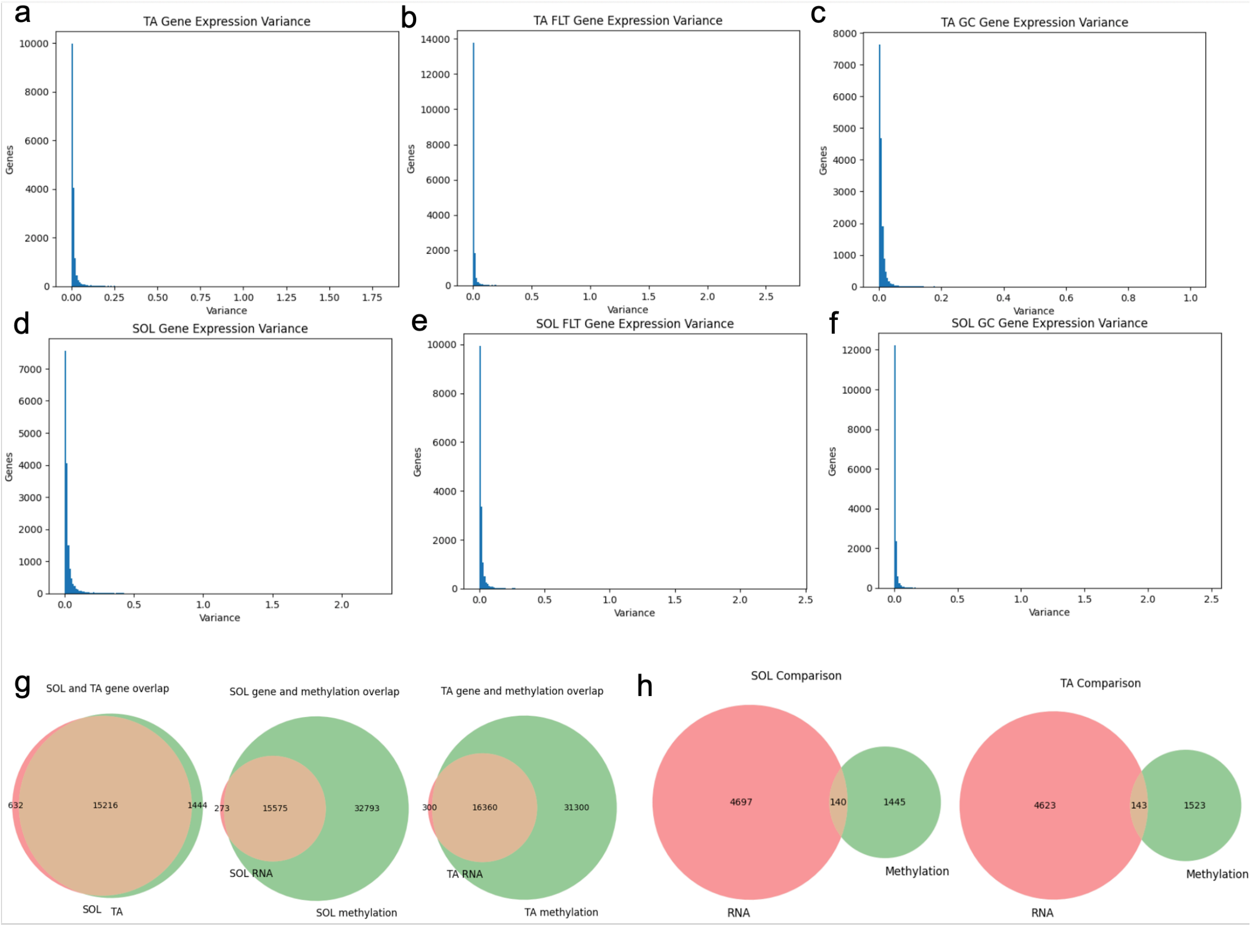

Supplementary Figure 5. Analysis of gene expression variance and overlap with methylation loci. a) Across-group TA gene expression variance distribution. **b)** Within-group TA FLT gene expression variance distribution. **c)** Within-group TA GC gene expression variance distribution. **d)** Across-group SOL gene expression variance distribution. **e)** Within-group SOL FLT gene expression variance distribution. **f)** Within-group SOL GC gene expression variance distribution. **g)** Overlap of genes present in SOL vs. TA gene expression, SOL gene expression vs. methylation, and TA gene expression vs. methylation. **h)** Overlap of the top 10% methylated genes and lowest 10% expressed genes in SOL and TA.

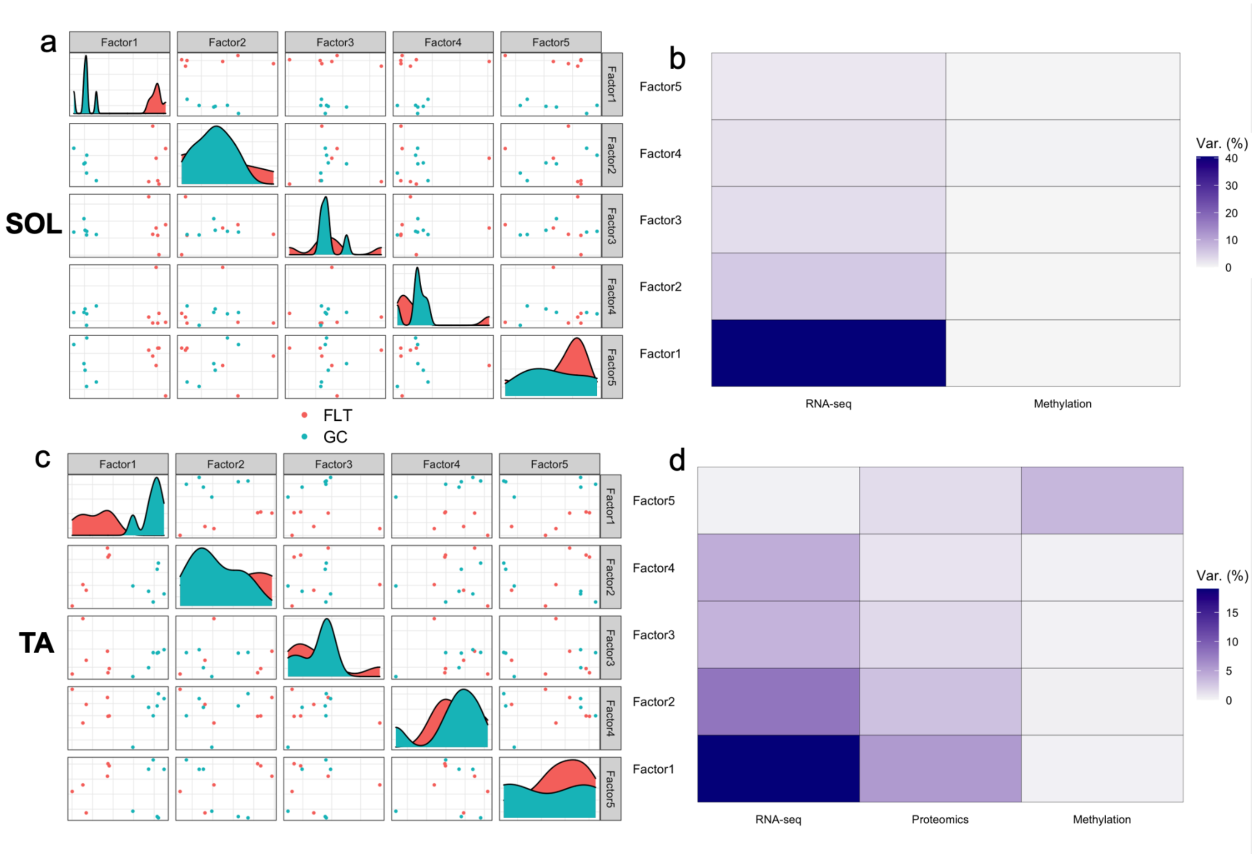

Supplementary Figure 6. MOFA factors contributions. a) Pair plots of the top 5 factors (by variance contribution) found by MOFA when using all available types of omics data for SOL. **b)** Contribution of each MOFA factor in explaining the % variance explained within each ome for SOL (OSD-104). **c)** Pair plots of the top 5 factors (by variance contribution) found by MOFA when using all available omes for TA. **d)** Contribution of each MOFA factor in explaining the percent variance explained within each ome for TA (OSD-105).

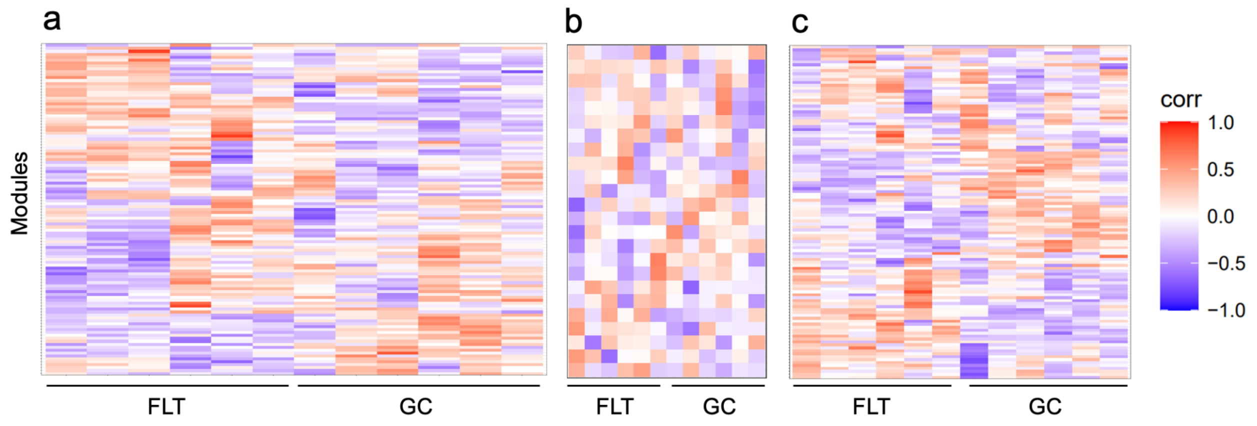

Supplementary Figure 7. WGCNA clusters. A) WGCNA clusters from TA RNA-seq data. **B)** WGCNA clusters from TA proteomics SOL data. **C)** WGCNA clusters from TA methylation data.

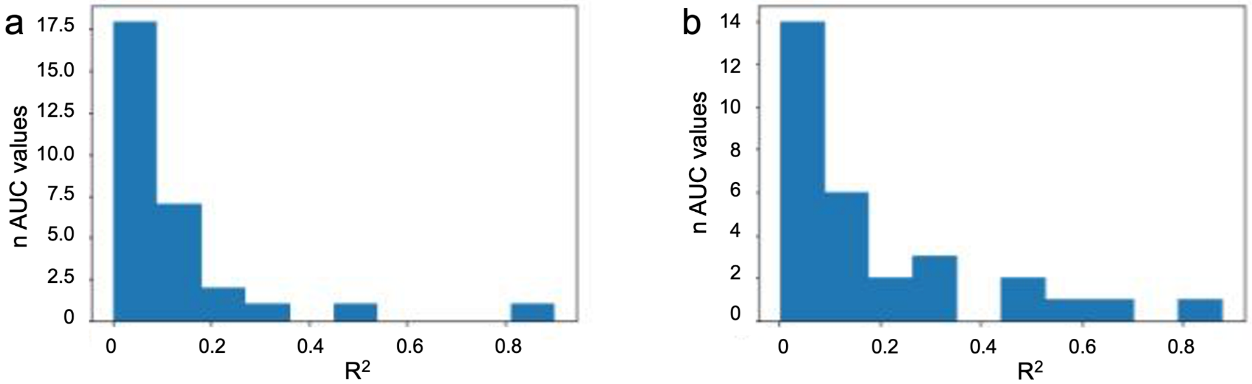

Supplementary Figure 8. Calcium labeling assignment distributions. The distribution of 30 random assignments of calcium AUC labels to **a)** OSD-104 SOL and **b)** OSD-105 TA mice. The x-axis is the resulting cross-validated R^2 scores when training QLattice with the labeling assignment.

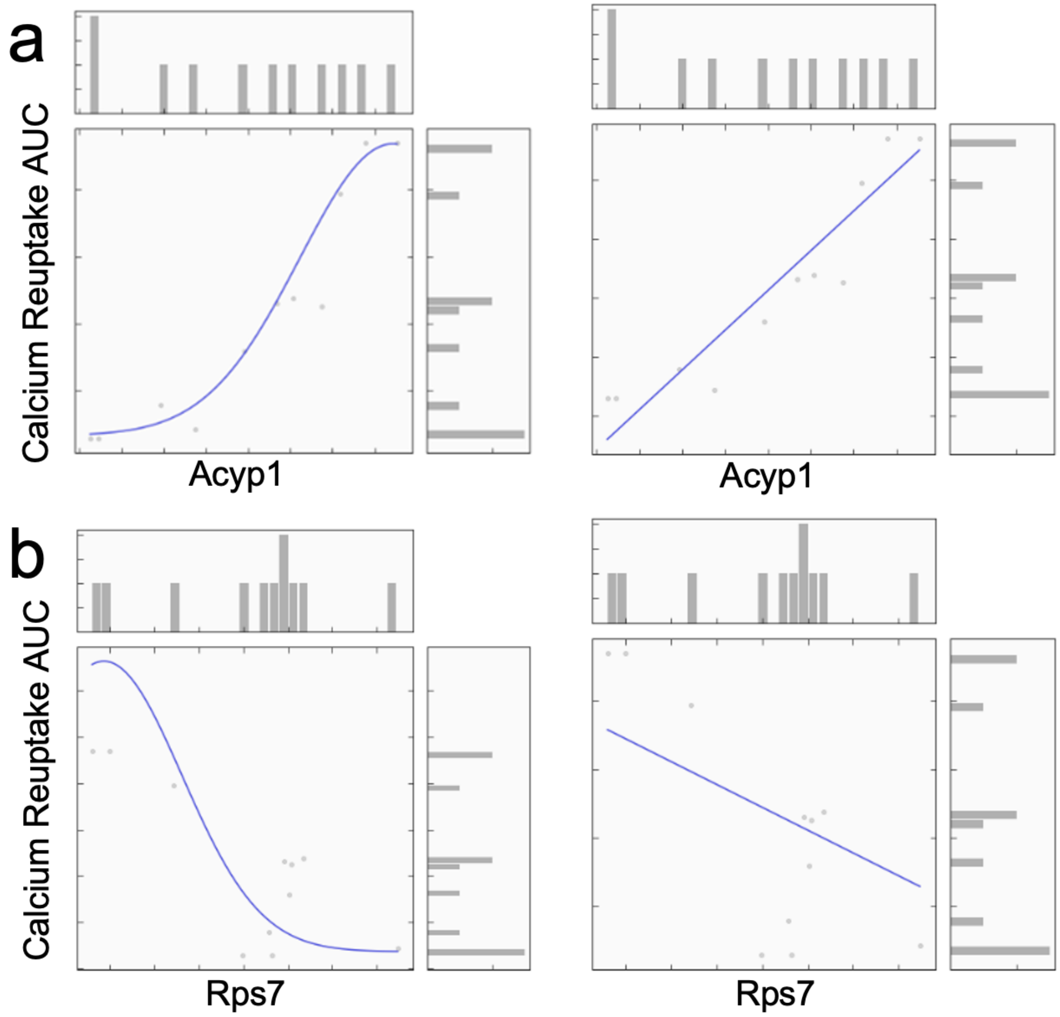

Supplementary Figure 9. Relationships between protein expression and calcium reuptake AUC in QLattice models. a) Representative examples of relationships between Acyp1 protein

and calcium reuptake AUC in QLattice models. **b)** Examples of relationships between Rps7 protein and calcium reuptake AUC.
